# HIF-1α effects on angiogenic potential in human small cell lung carcinoma

**DOI:** 10.1186/1756-9966-30-77

**Published:** 2011-08-15

**Authors:** Jun Wan, Huiping Chai, Zaicheng Yu, Wei Ge, Ningning Kang, Wanli Xia, Yun Che

**Affiliations:** 1 Department of Thoracic Surgery, the First Affiliated Hospital of Anhui Medical University, Hefei 230022, China

**Keywords:** SCLC, HIF-1α, chick embryo chorioallantoic membrane, angiogenesis

## Abstract

**Background:**

Hypoxia-inducible factor-1 alpha (HIF-1α) maybe an important regulatory factor for angiogenesis of small cell lung cancer (SCLC). Our study aimed to investigate the effect of HIF-1α on angiogenic potential of SCLC including two points: One is the effect of HIF-1α on the angiogenesis of SCLC *in vivo*. The other is the regulation of angiogenic genes by HIF-1α *in vitro *and *in vivo*.

**Methods:**

*In vivo *we used an alternative method to study the effect of HIF-1a on angiogenic potential of SCLC by buliding NCI-H446 cell transplantation tumor on the chick embryo chorioallantoic membrane (CAM) surface. *In vitro *we used microarray to screen out the angiogenic genes regulated by HIF-1a and tested their expression level in CAM transplantation tumor by RT-PCR and Western-blot analysis.

**Results:**

*In vivo *angiogenic response surrounding the SCLC transplantation tumors in chick embryo chorioallantoic membrane (CAM) was promoted after exogenous HIF-1α transduction (p < 0.05). *In vitro *the changes of angiogenic genes expression induced by HIF-1α in NCI-H446 cells were analyzed by cDNA microarray experiments. HIF-1α upregulated the expression of angiogenic genes VEGF-A, TNFAIP6, PDGFC, FN1, MMP28, MMP14 to 6.76-, 6.69-, 2.26-, 2.31-, 4.39-, 2.97- fold respectively and glycolytic genes GLUT1, GLUT2 to2.98-, 3.74- fold respectively. In addition, the expression of these angiogenic factors were also upregulated by HIF-1α in the transplantion tumors in CAM as RT-PCR and Western-blot analysis indicated.

**Conclusions:**

These results indicated that HIF-1α may enhance the angiogenic potential of SCLC by regulating some angiogenic genes such as VEGF-A, MMP28 etc. Therefore, HIF-1α may be a potential target for the gene targeted therapy of SCLC.

## Background

Hypoxia inducible factor-1 alpha (HIF-1α) is a member of the HIF-1 gene family, it is highly expressed in hypoxic conditions and degraded in normoxic condition [1,2]. HIF-1α activation is a common feature of tumors [3,4]; it is generally more pronounced in aggressive tumors [5] and can be an independent predictor of poor prognosis in certain types of cancer [6]. This is primarily due to the fact that HIF-1α plays a major role in the development of a characteristic tumor phenotype influencing growth rate, angiogenesis, invasiveness, and metastasis. Of these characteristics, angiogenesis is the most significant because it is essential for the other biological characteristics [7]. Several investigation about the angiogenesis of some kinds of malignant tumors such as breast and prostate cancer [8], head and neck cancer [9] have demonstrated that it is an intricate multistep and temporally ordered process that involves a great number of genes, modifiers and pathways regulated by HIF-1α. Some of these genes are directly induced by HIF-1α, such as NOS(nitric oxide synthases), angiogenic and vascular growth factors(VEGF) and urokinasetype plasminogen activator receptor (uPAR). Others are indirectly regulated by HIF-1α and might be influenced by secondary mechanisms. SCLC exhibits high expression levels of HIF-1α [10,11] and early hematogenous metastasis to other organs, such as brain, kidney, and liver, which relies on tumor angiogenesis [12]. However, the effect of HIF-1α on the angiogenic potential and regulation of angiogenic gene expression levels that influence this biological process have not been previously reported. In our study, we will use appropriate experimental methods to investigate these points.

For the *in vivo *study, we used the chick embryo chorioallantoic membrane (CAM) as the experimental model. CAM is an easily accessible and highly vascularized structure lining the inner surface of the egg shell that has been used to measure the invasive and angiogenic properties of tumor cell xenografts for the loss of the mature immune system in the early phase of development [13,14]. Several studies have investigated the formation of CAM vessels at different stages of development [15-17]. In this model, tumor cells are grafted to the CAM to reproduce the tumor characteristics *in vivo *including tumor mass formation, angiogenesis, and metastasis. Tumor explants and tumor cell suspensions have been shown to invade the chorionic epithelium and to form visible masses within 3 d to 5 d. After implantation and transplantation, the tumors can be macroscopically observed in the CAM [18]. Moreover, the growth and angiogenic responses of the transplantation tumors can be examined using microscopy and quantified for analysis. Therefore, the CAM model is an ideal model for cancer research [19,20].

With regard to the possible difference of growth and angiogenic responses after transduction by HIF-1α or siHIF-1α into SCLC cells, we think that HIF-1α may regulate the expression of some genes responsible for these biological characteristics. To identify these genes and confirm if HIF-1α influence the growth, invasiveness and angiogenesis of SCLC cells by up- or down-regulation of these genes involved in these activity, first we screened human gene chips containing 54614 unique cDNA clones using cDNA prepared from mRNA of SCLC cells in all the experimental groups. After these genes were screened out we continued to measure their expression levels in the xenografts formed by SCLC cells in the CAM by Transcriptase-polymerase chain reaction (RT-PCR) and Western-blot analysis. This study investigated the effect of HIF-1α on the angiogenic potential of the SCLC cells at histological, morphological, and molecular levels. Furthermore, this study demonstrated that HIF-1α may be used as a potential target for the treatment of SCLC in the future.

## Methods

### Cell culture and transduction with Ad5-HIF-1α and Ad5-siHIF-1α

**Figure 1:**
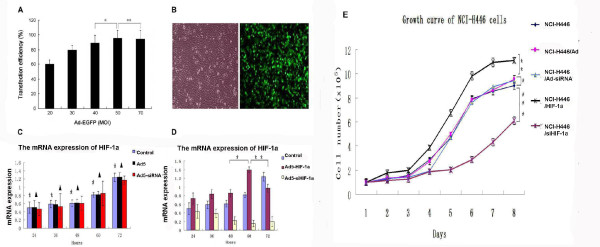
**Transduction of NCI-H446 cells with Ad5**. Chosing transduction condition and the effect on NCI-H446 cells growth by HIF-1α. (A)Five different multiplicities of infection (MOI: 20, 30, 40, 50, and 70) were tested in the transduction experiment (60 h). The transduction efficiency was the highest when the MOI was 50 (*p < 0.05 represents MOI50 vs. MOI40; **p < 0.05 represents MOI50 vs. MOI70). (B) Transduction efficiency of NCI-H446 cells with Ad5-EGFP after 60 h (MOI = 50; 200 ×). (C) After the cells were transduced with Ad5 and Ad5-siRNA(MOI = 50), the mRNA expression level of HIF-1α was measured in the indicated time period by real-time PCR (*p > 0.05 represents NCI-H446/Ad5 group vs control group; ^▲^p > 0.05 represents NCI-H446/Ad5- siRNA group vs control group;) (D)After the cells were transduced with Ad5-HIF-1α and Ad5-siHIF-1α (MOI = 50), the mRNA expression level of HIF-1α was measured in the indicated time period by real-time PCR (*p < 0.05 represents NCI-H446/HIF-1α group and NCI-H446/siHIF-1α group, 60 h vs. 48 h; ** p < 0.05 represents NCI-H446/HIF-1α group and NCI-H446/siHIF-1α group, 60 h vs. 72 h). (E) Growth curve of the cells in five groups. After transduction with Ad5 and Ad5-siRNA, the trendency of growth curve had no significant change. After transduction with HIF-1α, the growth curve of NCI-H446 cells shifted to the left with the growth of cells entering the period of logarithmic growth. After transduction with Ad5-siHIF-1α, however, the growth curve shifted to the right (*p > 0.05 represents NCI-H446/Ad5 or NCI-H446/Ad5-siRNA group vs. NCI-H446 group; **p < 0.01 represents NCI-H446/HIF-1α group vs. NCI-H446 group; ***p < 0.01 represents NCI-H446/siHIF-1α group vs. NCI-H446 group).

The NCI-H446 cell line was obtained from the American Type Culture Collection (ATCC; CAS; cell bank of Shanghai Institutes for Biological Sciences) and was cultured in RPMI-1640 medium (Sigma-Aldrich Co., St. Louis, MO, USA) supplemented with 10% fetal bovine serum (FBS; Hyclone) and 100-μg/ml kanamycin at 37°C in a humidified atmosphere containing 5% CO_2 _and 20% O_2_. The medium was routinely changed 2 d to 3 d after seeding. Cells were detached with trypsin/EDTA (GibcoBRL, Paisley, UK) and were resuspended in a 1:1 solution of serum-free RPMI-1640 medium to a final concentration of approximately 5 × 10^5 ^cells/10 μl. The appropriate transduction conditions of adenovirus (lengthen of time and multiplicity of infection-MOI) should be cleared for the analysis of microarry and PCR. The high transduction efficiency of Ad5 (a tumor-specific and replication-defective adenovirus used as the control vector) could reduce experimental error and resulted in differential expression levels of HIF-1α in Ad5-HIF-1α and Ad5-siHIF-1α treatment groups, which was favorable to investigate the effect of HIF-1α on the growth of NCI-H446 cells. We infected the cells by Ad5 and Ad5-siRNA and further eliminated the effect of adenovirus vector and non-targeting control siRNA. Ad5-EGFP, Ad5-siRNA-EGFP, Ad5-HIF-1α-EGFP and Ad5-siHIF-1α-EGFP adenoviruses were obtained from the Viral-Gene Therapy Department of Shanghai Eastern Hepatobiliary Surgery Hospital [21,22]. The sequences of the HIF-1α primers were as follows: upstream sequence (5'CTAGCTAGCTAGACCATG GAGGGCGGC'3) and downstream sequence (5'CGGGATCCTTATCAGTTAACTTGATC C'3). The sequences of the siHIF-1α primers were as follows: upstream sequence (5'TCGAG GAAGGAACCTGATGCTTTATTCAAGAGATAAAGCATCAGGTTCCTTCTTA'3) and downstream sequence (5'CTAGTAAGAAGGAACCTGATGCTTTATCTCTTGAATAAA GCATCAGGTTCCTTCC'3). As for Ad5-siHIF-1α, the pSilencer adeno 1.0-CMV system was purchased from Ambion for adenovirus construction. According to the manufacturer protocol deno-siHIF-1α was packaged and produced as the adenoviral backbone plasmid and the shuttle vector containing the siRNA template were linearized with PacI and then recombined in HEK-293 cells. After 10 days, Ad-siHIF-1α was obtained [22]. For the transduction experiments, cells were cultured in 6-well plates and were exposed to viral supernatants in the absence of cytokines and serum with different MOI. The titers of the Ad5-HIF-1α-EGFP and Ad5-siHIF-1α-EGFP adenoviruses were 1 × 10^10 ^pfu/L. Cytometry was used to calculate the cell number and the efficiency of transduction was estimated by determining the percentage of enhanced green fluorescence protein (EGFP)-positive cells. The appropriate MOI was chosed using the following formula: MOI = titer (pfu) × viral fluid (L)/cell number. When the MOI was 50, the transduction efficiency was more than 95% and expression was stable in a transduction experiment for 60 h (Figures 1A and 1B). In order to eliminated the effect of empty vector Ad5 and non-targeting control siRNA: Ad5-siRNA on HIF-1α mRNA expression and SCLC cells growth, transduction of NCI-H446 cells with Ad5 and Ad5-siRNA were carried out. In five selected time stages we found that empty vector Ad5 and Ad5-siRNA had no significant effect on the HIF-1α mRNA expression(Figure 1C). We selected the group(MOI = 50) for the high and stable transduction efficiency in the following experiments. HIF-1α mRNA levels in the NCI-H446 cells were measured by real-time PCR in our laboratory. The expression of HIF-1α mRNA was the highest in the Ad5-HIF-1α -treated cells and lowest in the Ad5-siHIF-1α-treated cells 60 h after transduction (Figure 1D). In addition, exogenous HIF-1α transduction significantly induced NCI-H446 cells growth and empty vector Ad5 and Ad5-siRNA transduction had no significant effect on the growth of NCI-H446 cells (Figure 1E).

### In vivo CAM assay

For the *in vivo *study, we used the CAM as an experimental vector to evaluate different tumor parameters. Four-day-old fertilized white leghorn chicken eggs (50 g-65 g) were incubated under 60% relative air humidity at 37°C and were rotated hourly with standing. On the third day of incubation, an irregular window (2 × 1.5 cm) was made on the top of the air chamber at the large, blunt end of the egg. A 21-gauge needle was used to puncture the endoconch membrane. Sterilized saline (0.1 ml) was administrated by injection to detach the endoconch membrane from the CAM. A second air chamber, called the flase air chamber (distinguished from the autospecific air chamber), was set up between these two membranes. The transduced and non-transduced cell suspensions (5 × 10^4 ^cells/μl) were gently pipetted onto the CAM surface with a transfer pipette. The eggs were then placed in the incubator. The engraftment growth was observed, and the tumor volume was calculated from day 4 to day 17 using the following formula: tumor volume (mm^3^) = (tumor length × width^2^)/2. The following three experimental groups that contained 12 samples each were used in this study: NCI-H446 group (control group), NCI-H446/Ad group, NCI-H446/Ad-siRNA group, NCI-H446/HIF-1α group, and NCI-H446/siHIF-1α group. The results were analyzed using a t-test and one-way ANOVA. The angiogenic responses were evaluated from day 8 to day 17 using a stereomicroscope connected to an image analyzer system in NCI-H446/Ad group (control group), NCI-H446/HIF-1α group, and NCI-H446/siHIF-1α group. Several parameters of angiogenesis, such as vessel area and number of vessel branches, were quantified by MIQAS quantified system analysis. For each study group, approximately 10 to 15 domains were selected for vessel quantification, and the mean values of the vessel number and vessel density were calculated.

### Histological assessment of transplantation tumors in the CAM

In order to identify the pathobiological characteristics of the transplantation tumors in the CAM, hematoxylin-eosin (HE) staining was used to evaluate the structure of the tumors and peripheral tissues. Neuron-specific enolase (NSE) is a specific marker of neuroendocrine tumor cells, such as SCLC cells, and is used as an important monitoring index in clinical diagnosis and therapy. Immunohistochemical analysis was performed to measure the expression of NSE. All tumor tissue sections from the paraffin blocks were deparaffinized, and endogenous peroxidases were inhibited with 0.3% hydrogen peroxide in methanol for 30 min. Antigen retrieval was achieved using 0.05% protease XIV at 37°C for 5 min. Sections were then incubated at room temperature for 1 h with a mouse anti-human NSE primary antibody (1:40 dilution; Wuhan Boster Biological Engineering Technology Co. Ltd.), rinsed with PBS, and incubated with a biotin-conjugated rabbit anti-mouse secondary antibody at room temperature for 45 min. The sections were subsequently incubated with a streptavidin-biotin-peroxidase complex (Vectastain ABC kit, Vector Laboratories, Burlingame, CA, USA) at room temperature for 45 min. The reaction was visualized using chromogen diaminobenzidine (DAB) for 10s. Sections were counterstained with haematoxylin, dehydrated, and permanently mounted.

### RNA extraction, microarray hybridization and data analysis

For the *in vitro *study, cDNA microarray technology was used to evaluate the change in the gene expression profile of NCI-H446 SCLC cells after transduction with Ad5-HIF-1α or Ad5-siHIF-1α and screened out the angiogenesis-related genes with differential expression. NCI-H446 cells were transduced with Ad5-HIF-1α or Ad5-siHIF-1α for 60 h. Afterwards, cells were washed with ice-cold phosphate-buffered saline (PBS) and lysed with 3 ml Trizol (Invitrogen, San Diego, CA, USA). Total RNA was extracted and purified using the RNAeasy kit according to the manufacturer's protocol (Qiagen, USA). The concentration of total RNA was measured with Biophotometer (Eppendorf, Germany) and the quality of purified RNA was confirmed by agarose gel electrophoresis. cDNA was then synthesized from each RNA sample using a SuperScript kit (Invitrogen), and the cDNA was used as a template for the preparation of biotin-labeled cDNA according to the GeneChip Labeling Kit protocol. The biotin-labeled cDNA was hybridized with a GeneChip (Human Genome U133 plus 2.0), washed, and stained with phycoerythrin-streptavidin according to the manufacturer's protocol. The microarray contained 54614 human gene probe sets, each of which consisted of 11 probe pairs corresponding to a single mRNA transcript. After saved as raw image files all the datas were converted into probe sets and analyzed by the software GCOS base on the method of normalization. Annotation by Unigene database http://www.ncbi.nlm.nih.gov/unigene, gene number, gene symbol and gene description were carried out using the database http://strubiol.icr.ac.uk/extra/mokca/ and Affymetrix databases [23]. The expression levels of angiogenic genes were presented as the ratio of the levels in the Ad5-HIF-1α group or Ad5-siHIF-1α group to the Ad5 control group. Ratio values greater than a 2-fold increase or decrease (p < 0.05) was considered to be significant expression changes. The primary data sets are all available at the following website: http://www.ncbi.nlm.nih.gov/gene

### Transcriptase-polymerase chain reaction (RT-PCR) analysis

We used RT-PCR to detect the expression of angiogenic genes obtained from microarray data in the transplantation tumor and CAM. On day 17 of incubation the angiogenic reaction reached the most intense level as explaining in the section of result, so we chosed the tumors of this day to detect. RT-PCR was performed using an RNA PCR kit (AMV) *ver *3.0 according to the manufacturer instructions (TaKaRa). Total RNA was extracted from transplantation tumor and CAM as described above. Level of mRNA expression of human and chicken angiogenic factors were evaluated by PCR using specific primers for human and chicken transcripts. The relative amount of the each PCR product was normalized to β-actin. Specific primers of these transcripts were designed by Primer Premier 5.0 (Table 1) and were synthesized by Shanghai Sangon Biological Engineering Technology & Services Co. The PCR program of angiogenic genes and β-actin consisted of 30 cycles of a denaturation step at 95°C for 30 seconds, an annealing step at 60°C for 30 seconds and an extension step at 75°C for 30 seconds followed by a final extension at 72°C for 5 minutes. PCR products were electrophoresed on a 1% agarose gel containing ethidium bromide. The band density was measured using the software Alpha Image 2000. The mRNA levels of the selected genes were normalized to β-actin to produce arbitrary units of relative transcript abundance.

**Table 1:** PCR reaction conditions and primer sequences

Gene	Primer	Tm(°C)	Length(bp)
Human			
VEGF-A	sense 5'-TGGAAGAAGCAGCCCATGAC-3'	59	375
	antisense 5'-GCACTAGAGACAAAGACGTG-3'		
IL-6	sense 5'-TCAATGAGGAGACTTGCCTG-3'	55	410
	antisense 5'-GATGAGTTGTCATGTCCTGC-3'		
PDGFC	sense 5'-GCCTCTTCGGGCTTCTCC-3'	56	395
	antisense5'-TTACTACTCAGGTTGGATTCCGC-3'		
FN1	sense 5'-CGAAATCACAGCCAGTAG-3'	51	278
	antisense 5'-ATCACATCCACACGGTAG-3'		
MMP28	sense 5'-CAAGCCAGTGTGGGGTCT-3'	56	252
	antisense 5'-TAGCGGTCATCTCGGAAG-3'		
MMP14	sense 5'-ATGTCTCCCGCCCCA-3'	60	678
	antisense 5'-TCAGACCTTGTCCAGCAGG-3'		
GLUT1	sense 5'-CGGGCCAAGAGTGTGCTAAA-3'	62	283
	antisense 5'-TGACGATACCGGAGCCAATG-3'		
GLUT2	sense 5'-CCTGAATGCCAAGGGAATCCGG-3'	48	368
	antisense 5'-GCCAGATGAGGTAATCAATCATAG-3'		
GAPDH	sense 5'-AGAAGGCTGGGGCTCATTTG-3'	57	258
	antisense 5'-AGGGGCCATCCACAGTCTTC-3'		
Chicken			
VEGF-A	sense 5'-GTCTACGAACGCAGCTTCTG-3'	62	265
	antisense 5'-TCACATGTCCAAGTGCGCAC-3'		
IL-6	sense 5'- TTGATGGACTCCCTAAGGC-3'	50	395
	antisense 5'-GATTCGGGACTGGGTTCTC-3'		
PDGFC	sense 5'-TTCTCAACCTGGATTCTGC-3'	52	355
	antisense 5'-AATGGTGTCAGTTCGCTTC-3'		
FN1	sense 5'-ACCAACATTGACCGCCCTAA-3'	56	458
	antisense 5'-AATCCCGACACGACAGCAGA-3'		
MMP28	sense 5'-TGACATCCGCCTGACCTT-3'	57	376
	antisense 5'-GTCCTGGAAGTGAGTGAAGACC-3'		
MMP14	sense 5'-CGTGTTCAAGGAGCGGTGGC-3'	61	114
	antisense 5'-TAGGCGGCGTCGATGCTGT-3'		
GLUT1	sense 5'-CACTGTTGTTTCGCTCTTCG-3'	42	316
	antisense 5'-AATGTACTGGAAGCCCATGC-3'		
GLUT2	sense 5'-AGTTTGGCTACACTGGAG-3'	60	436
	antisense 5'-AGGATGGTGACCTTCTCC-3'		
GAPDH	sense 5'-CTTTCCGTGTGCCAACCC-3'	65	108
	antisense 5'-CATCAGCAGCAGCCTTCACTAC-3'		

Tm - annealing temperature

Length - the number of bp in the PCR products

### Western blot analysis

On day 17 of incubation, the transplantation tumors and peripheral tissues of the CAM were harvested and homogenized in lysis buffer (50-mmol/L Tris, pH 7.4; 100-μmol/L EDTA; 0.25-mol/L sucrose; 1% SDS; 1% NP40; 1-μg/ml leupeptin; 1-μg/ml pepstatin A; and 100-μmol/L phenylmethylsulfonylfluoride) at 4°C. The protein was electrophoresed on SDS poly-acrylamide gels and transferred to a PVDF membrane. The membranes were then blocked at room temperature for 1 h with 5% non-fat milk in Tris-buffered saline containing Tween 20 (TBST) followed by incubation with rat anti-human and rat anti-chicken primary antibodies against VEGF-A (Wuhan Boster Biological Engineering Technology Co. Ltd.) overnight at 4°C. The membranes were subsequently incubated with goat anti-rat peroxidase- conjugated secondary antibodies. Immunoreactivity was detected by an enhanced chemiluminescence kit and was captured on X-ray film.

### Statistical analysis

All values were presented as means ± standard deviation (SD). The Student's t-test or one-way ANOVA was used to compare the parameters between the different study groups. P-values less than 0.05 were considered statistically significant. The statistical analyses were performed by the Windows SPSS 13.0 software.

## Results

### Implantation of cells on CAM *in vivo*

**Figure 2:**
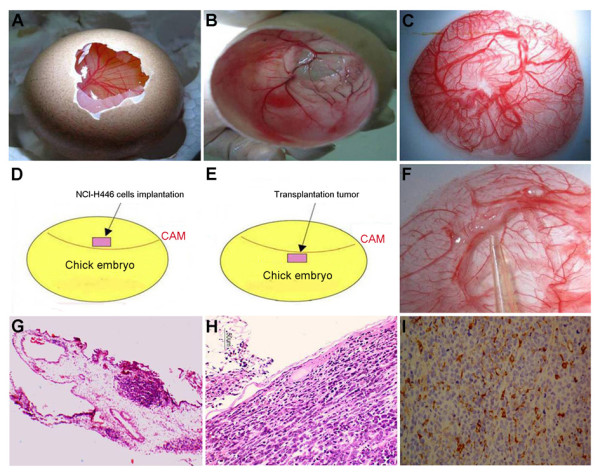
**Macroscopic examination of the CAM and implanted human NCI-H446 cells**. The entire experimental process from the implantation of NCI-H446 cells on the CAM and the formation of the transplantation tumor is shown. (A) Irregular window made in the egg shell of a 7-day-old chick embryo. (B) Elimination of the chick embryo in the CAM was observed. (C) The CAM was peeled for the assay. (D) Diagram of the technique for the implantation of NCI-H446 cells onto the CAM. (E) Diagram of the technique for the formation of the transplantation tumor. (F) The transplantation tumor (white mass was pointed by the tip) was formed on the side facing the chick embryo. (G-H) Histological evaluation of the transplanted tumor on the CAM by hematoxylin-eosin staining is shown:(G) The structure of the transplantation tumor and peripheral vessels (50 ×). (H) Pathological appearance of the transplantation tumor (200 ×). (I) Specific analysis was carried out by immunohistochemistry for the expression of NSE. The cellular nucleus was irregular, and positive expression for NSE was found in the intercellular substance or endochylema (400 ×).

The CAM was well-developed, and the vessels rapidly increased at day 7 (Figures 2A, B, and 2C). The NCI-H446 cell suspensions were implanted on the side of the CAM facing the window. The cell suspensions invaded across the capillary plexus and formed a visible mass on the side of the chicken embryo (Figures 2D and 2E). The chicken embryo tissue was eliminated, and the CAM with the transplantation tumor is shown in Figure 2F. The morphological and pathological characteristics of the tumor are shown in Figure 2G, and 2 its peripheral vessel is shown in Figure 2H. After sections were stained with an antibody specific for the human NSE protein, it was observed that the SCLC transplantation tumor cells were irregularly arranged, and that the nuclei were round or oval. Moreover, several tumor cells presented karyokinesis. Human NSE (shown by the yellow DAB stain) was distributed around the nucleus or in the intercellular space. In addition, human NSE expression was also observed around the vessel wall of the tumor (Figure 2I). As NSE is a specific marker of neuroendocrine tumor cells, such as SCLC cells, we verified that the transplantation tumor cells in the CAM were derived from SCLC.

**Figure 3:**
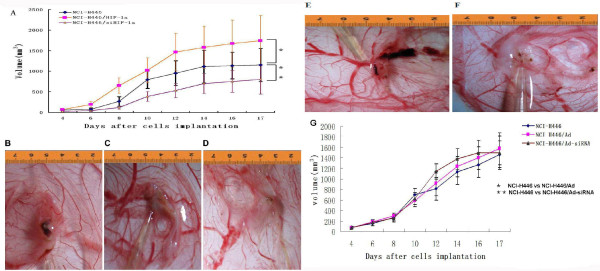
**Growth of the transplantation tumor**. The growth curves of the transplantation tumors in the three groups are shown. Data are presented as means ± SD. (A) The growth curves of transplantation tumors in the NCI-H446/HIF-1α group shifted left, and the growth curves shifted right in the Ad5-siHIF-1α group (*p < 0.01 represents NCI-H446/HIF-1α group vs. NCI-H446 group; **p < 0.01 represents NCI-H446/siHIF-1α group vs. NCI-H446 group). (B) A transplantation tumor from the NCI-H446 group (10 d after implantation). (C) A transplantation tumor from the NCI-H446/HIF-1α group (10 d after implantation). (D) A transplantation tumor from the NCI-H446/siHIF-1α group (10 d after implantation). (E) A transplantation tumor from the NCI-H446/Ad5 group (10 d after implantation). (F) A transplantation tumor from the NCI-H446/Ad5-siRNA group (10 d after implantation). (G) Comparing to the growth curves in NCI-H446 group the tendency of the curves in NCI-H446/Ad5 group and NCI-H446/Ad5-siRNA group had no significant changes. (*p > 0.05 represents NCI-H446 group vs. NCI-H446/Ad5 group; **p > 0.01 represents NCI-H446/Ad5-siRNA group vs. NCI-H446 group).

Chick embryo death was determined by the matte appearance of the CAM and yolk sac. The survival rate of chick embryos after the implantation of cells without transduction onto CAM was 92.5% (74 of 80), and the survival rate of chick embryos after implantation of cells transduced with Ad5-HIF-1a was 81.25% (65 of 80). Moreover, the chick embryo survival rate after the implantation of cells transduced with Ad5-siHIF-1a was 91.25% (73 of 80). Diffuse patches of NCI-H446 cells were observed in the CAM by the third day after implantation, but tumors were not large enough to be accurately measured until the fourth day in all three experimental groups. As shown in Figure 3A, the tumors in the HIF-1α transduction group grew more rapidly when compared to the control group (p < 0.01). The tumors in the siHIF-1α transduction group grew slower than the control group (p < 0.01). This result was in agreement with the growth of NCI-H446 cells *in vitro*. The same circumstance was presented from the three growth curves showing that tumor volume increased nearly exponentially from day 4 to day 10 but slowly from day 14 to day 17 as the growth curves became flat. This data suggests that more mature immune systems inhibited the tumor growth to some extent. With regard to angiogenesis, the vessels in the NCI-H446/HIF-1α group were larger and more dense (Figure 3C) when compared to the peripheral vessels around the tumors in the NCI-H446 group (Figure 3B). However, the vessels in the NCI-H446/siHIF-1α group were less dense (Figure 3D) when compared to the peripheral vessels around the tumors in the NCI-H446 group (Figure 3B). Beside these we also compared the transplantation tumors between NCI-H446 group, NCI-H446/Ad group(Figure 3E) and NCI- H446/Ad-siRNA group(Figure 3F) and no significant difference could be found in the angiogenic reaction between three groups. We also found that empty adenovirus vector and non-targeting control siRNA transduction had no significant effect on the growth of tumors(Figure 3G).

**Figure 4:**
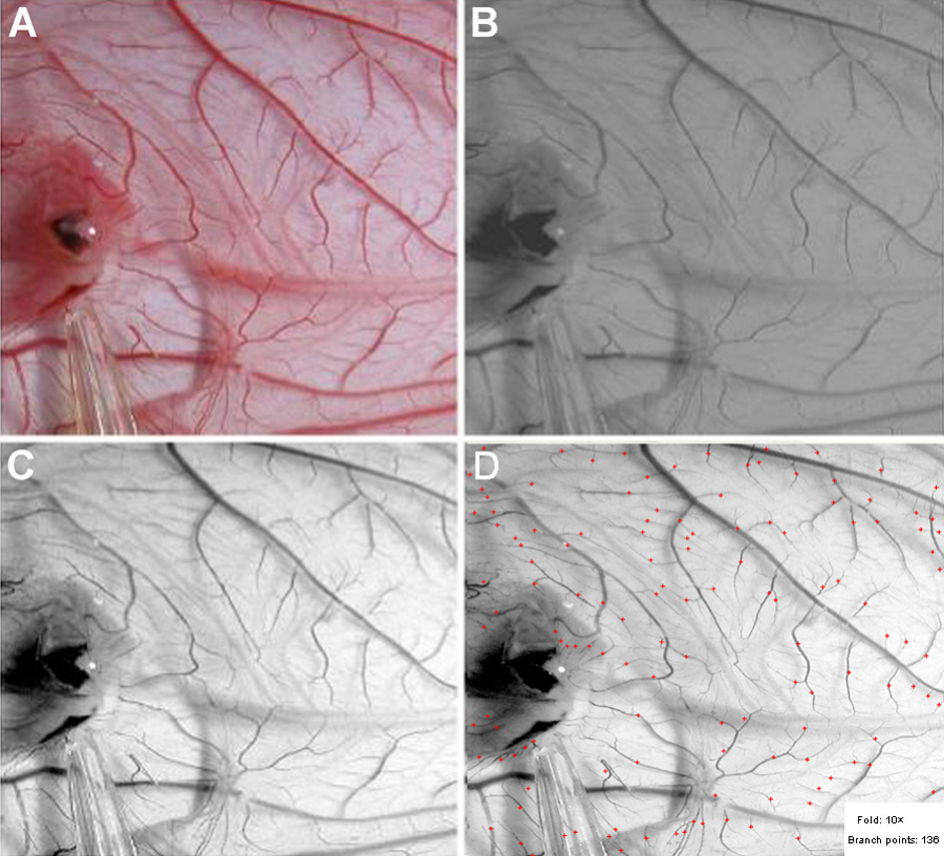
**Angiogenesis quantification of CAM**. The entire process of angiogenesis quantification on the CAM was divided into four steps. (A) The image of one special domain in the CAM was collected for the assay. (B) The background of the image was cleaned up. (C) The profiles of the vessels for the assay were deepened. (D) The result of the MIQAS quantified system analysis for the number of vessel branch points as marked by the red points.

The angiogenic image was captured (Figure 4A) and converted to grayscale (Figure 4B). We then eliminated the background of the graph (Figure 4C) and marked the vessels for quantification (Figure 4D). Our results indicated that on day 17 of incubation the angiogenic reaction reached the most intense level. NCI-H446 cells stimulate angiogenesis and the cells transduced with HIF-1α significantly promote the angiogenic effect. In contrast, the blockade of HIF-1α by Ad5-siHIF-1α inhibited the angiogenic effect (Table 2). In addition we also found that two parameters showed the similar increasing trends along with the growth of transplantation tumor and the time of transduction by HIF-1α (Table 2).

**Table 2:** Quantification of vessel area and the number of vessel branches around the transplantation tumor

	day 8	day 11	day 14	day 17
Vessel length (pixels)				
Control (n = 10 × 4)	2106 ± 143	1967 ± 113	1457 ± 135	2183 ± 156
NCI/H446(n = 10 × 4)	2452 ± 117	2564 ± 96*	2687 ± 103*	2798 ± 135*
NCI/H446/HIF-1α(n = 15 × 4)	2742 ± 83	2814 ± 154	2910 ± 137^§^	2994 ± 124^§^
NCI/H446/siHIF-1α(n = 12 × 4)	2331 ± 53^#^	2268 ± 106^#^	2236 ± 162^#^	2203 ± 116^#^

Vessel Branch points				
Control (n = 10 × 4)	76 ± 5	82 ± 9	73 ± 8	89 ± 5
NCI/H446(n = 10 × 4)	92 ± 7	101 ± 11	105 ± 6*	117 ± 7*
NCI/H446/HIF-1α(n = 15 × 4)	116 ± 16	123 ± 11^§^	128 ± 9^§^	134 ± 21^§^
NCI/H446/siHIF-1α(n = 12 × 4)	82 ± 5^#^	87 ± 6^#^	92 ± 11^#^	102 ± 13^#^

The MIQAS quantified system was used for the quantification of the two vessel parameters around the transplantation tumor in the CAM. Data are presented as means ± SD.

*Significant difference from group controls at p < 0.05 by use of paired sample t-test

^§^Significant difference from group controls at p < 0.05 by use of one-way ANOVA

**^#^**significant difference from group controls at p < 0.05 by use of one-way ANOVA

### Regulation of angiogenic gene expression by HIF-1α

To evaluate the effect of HIF-1α on the gene expression profile, we used the comparative analysis algorithm provided by Genespring to compare the effect of HIF-1α among the three groups (Ad5, Ad5-HIF-1α, and Ad5-siHIF-1α). Among the genes with differential expression (more than 2 fold), we selected 15 genes (Table 3) associated with angiogenesis. We found that VEGF-A, which is a known target gene of HIF-1α, was significantly increased by more than 6 fold after transduction by Ad5-HIF-1α and reduced by approximately 4 fold after transduction by Ad5-siHIF-1α. HIF-1α also increased the expression of several inflammatory factors, such as interleukin 6 (IL6), tumor necrosis factor alpha-induced protein 6 (TNFAIP6), and interleukin 1 receptor type I (IL1RI). These results indicated that angiogenesis in SCLC induced by HIF-1α may be related to inflammatory responses because the expression levels of several corresponding inflammatory factors were upregulated. Matrix metalloproteinase-28 (MMP-28) and matrix metalloproteinase-14 (MMP-14) are important members of the MMP family, and matrix degradation is the precondition of angiogenesis in tumors. The upregulation of MMP-28 and MMP-14 indicated that HIF-1α may promote matrix degradation to induce angiogenesis in SCLC. HIF-1α also induced other angiogenic factors, such as tenascin C (TNC), platelet derived growth factor C (PDGFC), fibronectin 1 (FN1), myocardin (MYOCD), and heme oxygenase decycling 1 (HMOX1). In contrast, HIF-1α decreased the expression levels of the following genes: suppressor of cytokine signaling 2 (SOCS2), insulin-like growth factor binding protein 3 (IGFBP3), insulin-like growth factor 1 receptor (IGF1R), and cysteine-rich angiogenic inducer 61 (CYR61). The most significant downregulation of gene expression was found in the SOCS2 gene. Besides these, two glycolytic genes glucose transporter 1(GLUT1) and glucose transporter 2 (GLUT2) were upregulated by HIF-1α to 2.98 and 3.74 respectively, so we concluded that HIF-1α maybe upregulate the glycolysis reaction of SCLC.

**Table 3:** The effect of HIF-1α on angiogenic gene expression

UniGeneID	Gene name	Gene Symbol	Fold change (ratio ≥ 2, p < 0.05)	
			**A**	**B**

Hs.143250	Tenascin C (hexabrachion)	TNC	5.28	-3.23
Hs.654458	Interleukin 6 (interferon, beta 2)	IL6	5.29	-2.27
Hs.73793	Vascular endothelial growth factorA	VEGF-A	6.76	-3.98
Hs.437322	Tumor necrosis factor, alpha-induced protein 6	TNFAIP6	6.96	-4.75
Hs.570855	Platelet derived growth factor C	PDGFC	2.26	-3.21
Hs.701982	Interleukin 1 receptor, type I	IL1R1	2.64	-2.21
Hs.203717	Fibronectin 1	FN1	2.31	-2.57
Hs.567641	Myocardin	MYOCD	3.03	-2.08
Hs.517581	Heme oxygenase (decycling) 1	HMOX1	2.64	-2.73
Hs.687274	Matrix metallopeptidase 28	MMP28	4.39	-3.67
Hs.2399	Matrix metallopeptidase 14	MMP14	2.97	-2.24
Hs.473721	Glucose transporter 1	GLUT1	2.98	-2.16
Hs.167584	Glucose transporter 2	GLUT2	3.74	-2.05
Hs.485572	Suppressor of cytokine signaling 2	SOCS2	-6.06	3.06
Hs.450230	Insulin-like growth factor binding protein 3	IGFBP3	-4.02	2.17
Hs.653377	Insulin-like growth factor 1 receptor	IGF1R	-2.00	2.89
Hs. 8867	Cysteine-rich, angiogenic inducer, 61	CYR61	-3.03	2.18

cDNA microarray analysis was used to screen angiogenic genes with differential expression (more than 2.0-fold) between the following two comparison groups: Ad5 vs. Ad5-HIF-1α and Ad5 vs. Ad5-siHIF-1α.

A = Ad5 vs. Ad5-HIF-1α; 11 genes were upregulated and 4 genes were downregulated by HIF-1α

B = Ad5 vs. Ad5-siHIF-1α; 4 genes were upregulated and 11 genes were downregulated by siHIF-1α (contrasting the A group)

### RT-PCR analysis for angiogenic factors in CAM

**Figure 5:**
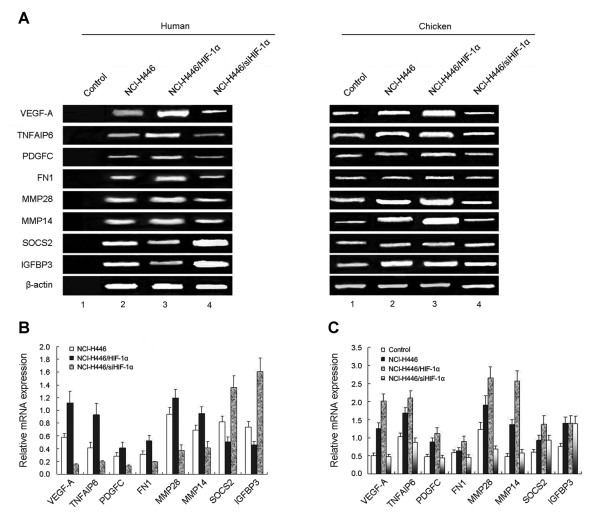
**RT-PCR analysis of human and chicken angiogenic factors mRNA**. Microarray analysis was performed to screen out the angiogenic factors affected by HIF-1α in SCLC cells (table 2). Afterwards, RT-PCR analysis was used to detect the expression of angiogenic factors affected by HIF-1a in the transplantation tumors of CAM *in vivo*. (A), Human and chicken VEGF-A, TNFAIP6, PDGFC, FN1, MMP28, MMP14, SOCS2 and IGFBP3 mRNA expression: Representative images of three independent experiments (Lane 1: control group-no human mRNA expression, Lane 2: transplantation tumor of NCI-H446 cells transduction by empty vector Ad5-NCI-H446 cells group, Lane 3: ransplantation tumor of NCI-H446 cells with transduction by HIF-1α-NCI-H446/HIF-1α group, Lane 4: transplantation tumor of NCI-H446 cells with transduction by siHIF-1α-NCI-H446/siHIF-1α group). (B and C), Relative expression levels of mRNA in NCI-H446/HIF-1α group and NCI-H446/siHIF-1α group compared with that in control group and NCI-H446 cells group (p < 0.05).

**Figure 6:**
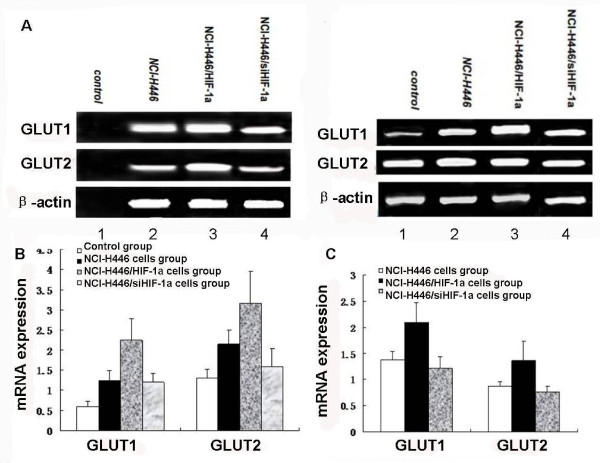
**RT-PCR analysis of human and chicken glycolytic factors mRNA**. RT-PCR analysis was used to detect the expression of glycolytic factors affected by HIF-1a in the transplantation tumors of CAM *in vivo*. (A), Human and chicken GLUT1 and GLUT2 mRNA expression: Representative images of three independent experiments (Lane 1: control group-no human mRNA expression, Lane 2: transplantation tumor of NCI-H446 cells transduction by empty vector Ad5-NCI-H446 cells group, Lane 3: ransplantation tumor of NCI-H446 cells with transduction by HIF-1α-NCI-H446/HIF-1α group, Lane 4: transplantation tumor of NCI-H446 cells with transduction by siHIF-1α-NCI-H446/siHIF-1α group). (B and C), Relative expression levels of mRNA in NCI-H446/HIF-1α group and NCI-H446/siHIF-1α group compared with that in control group and NCI-H446 cells group (p < 0.05).

We used RT-PCR analysis to study the angiogenic potential of NCI-H446 SCLC cell implanted on the CAM. We found that HIF-1a increased mRNA expression levels of human and chicken VEGF-A, TNFAIP6, PDGFC, FN1, MMP28, MMP14(Figure 5A-C) GLUT1, GLUT2 (Figure 6A-C), but decreased the expression of human SOCS2 and IGFBP3. However, no changes in the expression of chicken angiogenic factors SOCS2 and IGFBP3 were observed in transplantation tumors of CAM (Figure 5A-C).

### Western blot analysis for VEGF-A expression

**Figure 7:**
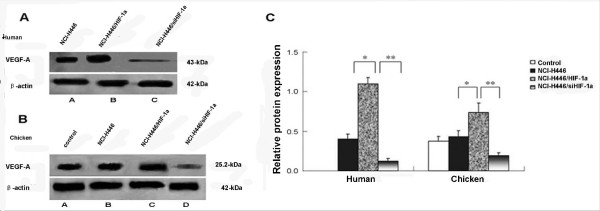
**Western blot analysis of the human and chicken VEGF-A protein in the CAM**. In the NCI-H446/HIF-1α and NCI-H446/siHIF-1α groups, the SCLC cells were transduced with Ad-HIF-1α or Ad-siHIF-1α (MOI = 50) for 60 h before implanting onto the CAM to form transplantation tumors. Western blots were performed to detect the VEGF-A protein level in the tumors and peripheral tissues on day 17 of incubation. Data are presented as means ± SD. (A) Representative images of three independent experiments (Lane A - human VEGF-A protein expression in the tumors from the NCI-H446 group; Lane B - human VEGF-A protein expression in the tumors from the NCI-H446/HIF-1α group; and Lane C - human VEGF-A protein expression in the tumors from the NCI-H446/siHIF-1α group) (human - * p < 0.05 group C vs. group B; ** p < 0.05 group C vs. group D) (chicken - * p < 0.05 group C vs. group B; ** p < 0.05 group C vs. group D). (B) Representative images of three independent experiments (Lane A - chicken VEGF-A protein expression of control group; Lane B - chicken VEGF-A protein expression in the tumors from the NCI-H446 group; Lane C - chicken VEGF-A protein expression in the tumors from the NCI-H446/HIF-1α group; and Lane D - Chicken VEGF-A protein expression in tumors from the NCI-H446/siHIF-1α group). (C) Densitometry analysis of the relative expression of VEGF-A protein compared to the corresponding β-actin in each group (p < 0.05).

VEGF is regarded as the gold standard of angiogenesis, and it has the most important role in the angiogenic process in tumors. VEGF-A is a member of the VEGF family, and it is a target gene of HIF-1α. In this study, both human and chicken VEGF-A protein expression levels were high in the CAM tissue of the HIF-1α transduction group as compared to the other groups (Figures 7A, B, and 7C). Similar to the real-time PCR results, we presumed that angiogenesis in the CAM induced by the transplantation tumor was affected by human VEGF-A to a greater extent than by chicken VEGF-A.

## Discussion

### Gene transduction of SCLC cells by HIF-1α

With regard to SCLC, a common pulmonary solid tumor, angiogenesis regulated by HIF-1α may have an important role in determining tumor phenotypes. In order to recapitulate the effect of HIF-1α in a hypoxic environment, we overexpressed human HIF-1α in SCLC NCI-H446 cells with the gene vector Ad5-based transduction system. The type 5 adenovirus-based transduction system is a transient expression system that allows protein expression in transduced cells to reach a higher level than the level found in non-transduced cells in a short period of time, which can reduce the possibility of experimental error to some extent [24]. According to our previous study, we used the appropriate plaque-forming unit (pfu) (MOI = 50) for a high expression level of HIF-1α [23] in this study. A gene-specific siRNA, which exhibited stronger suppressive effects than antisense oligonucleotides [25], was used to silence the expression of HIF-1α and to further confirm the effects of HIF-1α on NCI-H446 cells and transplantation tumors. The *in vitro *study demonstrated that cells transduced with HIF-1α grew more rapidly than control cells, and cells transduced with siHIF-1α grew more slowly than control cells. The *in vivo *study indicated that the tumor formation rate of the HIF-1α transduction group was significantly higher than the rate of the non-transduction and siHIF-1α transduction groups. Moreover, the average tumor growth rate in the HIF-1α gene transduction group was higher than the tumor growth rates in the non-transduction and siHIF-1α groups. Thus, these results suggest that HIF-1α may be involved in promoting the progression of SCLC. Our study further supports the previous opinion that HIF-1α is correlated with the development of an aggressive phenotype in some tumor models [26], and that HIF-1α has been identified as a positive factor for tumor growth [27].

### Induction angiogenesis of SCLC cells on CAM by HIF-1α

Chicken embryos are immunodeficient during embryonic development until day 19 of incubation [13]. Thus, CAM was first adapted by many investigators as a convenient model to evaluate many different parameters of tumor growth [28] and to screen antineoplastic drugs [29,30]. Furthermore, the CAM model is an ideal alternative to the nude mouse model system for cancer research because it can conveniently and inexpensively reproduce many tumor characteristics *in vivo*, such as tumor mass formation, tumor-induced angiogenesis, infiltrative growth, and metastasis [31]. This model is especially ideal to study tumor-induced angiogenesis because of its dense vascular net and rapid vascular reactivity [32]. In this study, we have successfully established the transplantation tumor model and have clearly shown that the avian microenvironment provided the appropriate conditions for the growth of human SCLC cells, as in the case when they are transplanted into immunodeficient mice [33]. Moreover, the stroma of the CAM may represent a supportive environment for SCLC expansion because morphologically we could see that the SCLC cells were implanted on the side facing the window, invaded across the capillary plexus and formed a visible mass on the side of the chicken embryo.

With regard to targeted therapy of solid tumors, it is important to find a therapeutic target that is widely involved in many biological processes. HIF-1α is overexpressed in many human cancers. Significant associations between HIF-1α overexpression and patient mortality have been shown in cancers of the brain, breast, cervix, oropharynx, ovary, and uterus [2,4]. However, some scholars have suggested that the effect of HIF-1α overexpression depends on the cancer type. For example, associations between HIF-1α overexpression and decreased mortality have been reported for patients with head and neck cancer [34] and non-small cell lung cancer [35]. In our study, however, HIF-1α overexpression by Ad-HIF-1α significantly enhanced the angiogenic and invasive potential of SCLC, but transduction with Ad-siHIF-1α inhibited these potentials. Angiogenesis in SCLC is a key biological characteristic and an important mediator of tumor growth rate, invasiveness, and metastasis. Thus, the inhibition of angiogenesis is an effective method for the treatment of SCLC, and many targeted therapy drugs against angiogenesis, such as bevacizumab [36], cedirnnib [37], and sorafenib [38], have widely been used in clinical practice. However, the therapeutic targets of these drugs are confined to VEGF-A and its receptor or signaling pathway. VEGF-A is a downstream target of HIF-1α, and it contains HREs with an HIF-1α binding site [39]. In our study, the expression of VEGF-A and the vascular reaction in the transplantation tumor was significantly inhibited after the expression of HIF-1α was downregulated by siHIF-1α. In addition to VEGF-A, there are many angiogenic factors that are directly or indirectly regulated by HIF-1α. Therefore, we propose that targeting HIF-1α may provide a broader inhibition of tumor angiogenesis than targeting downstream angiogenesis factors of HIF-1α. In the future, we will conduct correlated research to confirm this proposal.

### Angiogenic factors regulated by HIF-1α in SCLC cells transplantation tumor

In pervious study although the multitude of insights were put into individual molecular effect on angiogenesis, such as increased migration and tube formation, which may be predicted to induce angiogenesis in vitro, these analyses in isolated systems clearly have their limitations, especially when a large scale of interconnections and complexity involved in the process of angiogenesis in vivo are considered. Allowing for this the *in vivo *expression of angiogenesis genes selected from the *in vitro *microarray analysis must be confirmed. Thus, it is important to successfully establish a simple and comprehensive model to test how HIF-1α regulates angiogenesis genes. Some scholars have suggested that xenograft models of tumor cells rely more on angiogenesis than naturally occurring tumors and that the extent of angiogenesis is dependent on the site of implantation of the xenografts [40]. CAM is essentially a respiratory membrane with a dense vascular net that maintains the blood-gas exchange. For abundant blood supply and a special anatomical position in the chick embryo, the CAM may provide more precise and convincing data for angiogenic factors than other *in vivo *experimental models [31].

Recent research and development for a targeted drug for SCLC has focused on inhibiting the expression of angiogenic factors, such as VEGF-A. However, the microenvironment of SCLC cell growth is largely hypoxic, and HIF-1α is the primary regulatory factor for angiogenesis. The factors that are mediated by HIF-1α and involved in angiogenesis of SCLC have not been previously reported. Therefore, in our study, we initially evaluated the effects of HIF-1α on the invasiveness of SCLC, which precedes angiogenesis. Matrix metalloproteinases (MMPs) are a family of enzymes responsible for remodeling the extracellular matrix during growth and morphogenetic processes, which are important for tumor invasiveness. In our study, two members of the MMP family, MMP-14 and MMP-28, had increased expression resulting from HIF-1α overexpression in the *in vitro *microarray experiment and in the CAM experiments. The increased expression of MMP-14 has been identified as a negative predictor of survival in SCLC [41], and the targeted drug inhibiting MMP-14 expression, marimastat [42], has been used in clinical studies. MMP-28 is expressed at low levels in normal lung tissue, but the expression of MMP-28 is highly increased after cancer formation [43]. MMP-28 induces epithelial-mesenchymal transitions (EMT), which yield tumor cells with collagen-invasive properties allowing the invasion of collagen matrices [44]. The upregulation of MMP-28 by HIF-1α enhances this ability.

The expression level of angiogenic factors is the gold standard to measure the angiogenic potential of tumors, and the inhibition of the expression of angiogenic factors is the primary treatment for SCLC. Angiogenic factors that are significantly regulated by HIF-1α in a hypoxic microenvironment are also therapeutic target points [45]. In addition to VEGF, FGF-2 [46], ANG-2 [47], HIF-2α [48], and PDGFC are also involved in tumor angiogenesis. In this study, three inflammatory factors, IL-6, TNFAIP6, and IL1R1, were upregulated by HIF-1α. These inflammatory factors actively responded during the process of inflammatory angiogenesis. TNFAIP6 is the stimulating factor for TNF-α [49], and IL-1R1 is the receptor for IL-1 [50]. IL-6 and VEGF-A have synergistic effects in stimulating the proliferation and invasiveness of tumors by promoting angiogenesis [51]. Our results indicate that HIF-1α may enhance the inflammatory reaction or stimulate the secretion of coherent inflammatory factors to promote the angiogenesis of SCLC, which highlights the importance of anti-inflammation for the treatment of SCLC as some scholars have suggested [52]. In addition, the TNC, FN1, and HMOX1 cytokines were screen out by microarray analysis. TNC is an extracellular matrix protein with angiogenesis-promoting activities, and it has specific functions in vessel formation [53]. FN1 has been shown to be an angiogenic cytokine involved in angiogenesis during several pathological processes, such as psoriasis, diabetic retinopathy, and cancer [54]. The overexpression of HMOX1 has been observed in liver cancer [55], pancreatic cancer [56], and melanomas [57]. Targeting these cytokines for gene therapy of SCLC in the future requires their verification in clinical trials.

## Conclusions

Overall, our results suggest that HIF-1α significantly promotes the growth and angiogenesis of NCI-H446 cells by upregulating the expression of angiogenic genes. Moreover, our use of the chick CAM as an *in vivo *experimental model further confirms the expression of these genes induced by HIF-1α. Tumor growth on the chick CAM after they were grafted with human SCLC NCI-H446 cells represents an excellent model to study human SCLC angiogenesis. This study suggests that HIF-1α may be a potential target in the treatment of SCLC. In the future, we will further investigate human SCLC progression and invasiveness, and we will screen anti-angiogenic molecules in the CAM model to further enhance the number of possible genes for SCLC targeted therapies.

## Competing interests

The authors declare that they have no competing interests.

## Authors' contributions

JW carried out the molecular genetic studies, participated in sequence alignment and drafted the manuscript. HC conceived of the study and participated in its design. ZY participated in its design. WG carried out the RT-PCR assay. NK carried out the HE staining and Western-blotting assay. WX helped to carried out microarray. YC participated in the design of study. All authors read and approved the final manuscript.
